# Assessing the innovation competency and entrepreneurial capacity of health students in Vietnam: a cross-sectional study

**DOI:** 10.1186/s12909-026-08928-y

**Published:** 2026-03-17

**Authors:** Anh Ngoc Thi Le, Phong Hoang Nguyen, Thao Phuong Truong, Linh Phuong Le, Trang Thi Nguyen, Ernest Guevarra, Proochista Ariana, Minh Cong Tran

**Affiliations:** 1 Genomica Vietnam Co., Ltd, Hanoi, Vietnam; 2 College of Health Sciences, VinUniversity, Hanoi, Vietnam; 3 Hanoi Medical University, Hanoi, Vietnam; 4 International University VNUHCM, Ho Chi Minh City, Vietnam; 5 Franklin and Marshall College, Lancaster, PA USA; 6 Nuffield Department of Medicine, International Health and Tropical Medicine, University of Oxford, Oxford, UK; 7 School of Computing and Mathematical Sciences, University of Leicester, Leicester, United Kingdom; 8 Hanoi Medical University Hospital, Hanoi, Vietnam

**Keywords:** Innovation, Entrepreneurship, Medical education, Health students, Vietnam

## Abstract

**Background:**

Innovation competency and entrepreneurial capacity have emerged as essential factors for future healthcare professionals in the context of digital transformation and complex patient demands. While global medical education has incorporated innovation training, research on innovation competency and entrepreneurial capacity among Vietnamese health students and related factors remains limited.

**Objectives:**

This study aims to assess the current level of innovation competency and entrepreneurial capacity among Vietnamese health students and identify factors associated with their development.

**Method:**

A cross-sectional study was conducted from May 2025 to July 2025, involving 370 students from various health-related majors across Vietnam. Participants completed an innovation competency scale based on Chell & Athayde’s (2009) five domain model and a validated entrepreneurial capacity questionnaire, developed based on Turker’s (2009) methodological proposal for scale development. The innovation competency scale includes creativity, leadership, energy, self-efficacy, and risk propensity, while the entrepreneurial capacity questionnaire consists of 4 domains of entrepreneurial capacity: management and business competency, entrepreneurial capacity, human resources competency, and interpersonal competency. Data were collected via an online survey and analysed using RStudio software, applying descriptive statistics, t-tests, ANOVA and regression.

**Results:**

The mean scores of participants’ innovation competency and entrepreneurial capacity were 137.06/217 (SD = 28.91) and 173.40/280 (SD = 35.20), respectively. Significant differences in innovation competency were observed across gender (*p* = 0.044), academic year (*p* < 0.001), major (*p* = 0.043), and geographic region (*p* = 0.005). Entrepreneurial capacity differed significantly by gender (*p* < 0.001) and academic year (*p* = 0.008). Linear mixed-effects model analysis revealed that participation in entrepreneurship courses, competitions, practice opportunities, and strong educational performance were positively associated with entrepreneurship education performance (*p* < 0.05).

**Conclusion:**

The findings contribute to existing research by offering one of the first integrated assessments of innovation competency and entrepreneurial capacity among health students in Vietnam amidst a rapidly transforming context of healthcare education. Overall, Vietnamese health students showed strong motivation and solid understanding in management and business; however, skills related to opportunity recognition, leadership, risk propensity, and communication remain limited. This suggests that current training emphasizes theory over practice. Educational interventions such as experiential learning, leadership training, and risk management workshops would contribute to innovation and entrepreneurial capacity among Vietnamese health students.

**Supplementary Information:**

The online version contains supplementary material available at 10.1186/s12909-026-08928-y.

## Introduction

Innovation and entrepreneurship have emerged as key drivers of progress in the healthcare sector, enabling the development of solutions to meet evolving patient needs and address systemic challenges. As healthcare systems around the world face increasing demands from ageing populations, rising healthcare costs, and emerging global health threats, the ability to innovate and deploy new technologies, services, and business models is essential [1]. Healthcare professionals equipped with innovation and entrepreneurial skills can make significant contributions to public health by identifying gaps in care delivery, optimising resources, and creating patient-centred solutions [2–4].

The Entrepreneurship Competence Framework defines entrepreneurial capacity as “when you act upon opportunities and ideas and transform them into value for others. The value that is created can be financial, cultural, or social” [5]. Entrepreneurial capacity in the healthcare sector is shaped by various factors that influence an individual’s ability to identify opportunities, develop innovations, and successfully implement new solutions. Personal traits such as risk tolerance, resilience, creativity, and proactiveness are essential for entrepreneurial success. Zhao and Seibert (2006) suggested that individuals with high levels of self-efficacy and openness to experience are more likely to engage in entrepreneurial activities [6]. Additionally, support mechanisms from authorities such as tax incentives, funding programs, and innovation hubs create an enabling environment for entrepreneurs to develop and scale healthcare solutions [7].

Innovation competency is defined as the ability to identify opportunities, generate novel ideas, and implement effective solutions; it is increasingly recognised as a core requirement for healthcare professionals in the 21st century [8, 9]. In the context of rapid technological advancement, digital health transformation, and complex patient needs, the capacity to innovate empowers future clinicians to lead meaningful change in health systems. Driven by the proliferation of smartphones, wearable technologies, and low-cost electronics, digital health is transforming care delivery and creating new opportunities for healthcare professionals to contribute to system innovation [10]. Recent studies have highlighted both opportunities and challenges when integrating digital and AI competencies into medical training, pointing to limited formal exposure among students and the need for coordinated national strategies to equip healthcare graduates with the skills required for digitally enabled practice. Clinician innovators, those who integrate clinical expertise with strategic thinking and design skills, are playing a growing role in addressing these emerging challenges [3, 4]. Recent evidence underscores that digital transformation in higher education and healthcare requires innovation competencies that extend beyond traditional skills, emphasizing digital literacy, collaboration, and problem solving [11, 12]. These contemporary perspectives reinforce the relevance of innovation competency as an essential outcome of modern medical education.

Some colleges and universities regard innovation and entrepreneurship education as an important starting point to deepen talent training reform and improve the quality of talent training. Integrating innovation and entrepreneurship into medical education training promotes critical thinking, adaptability, and problem-solving abilities, leading to advances such as telemedicine, personalised medicine, and AI-based diagnosis [4].

Fueled by rapid economic development, Vietnamese medical universities are also undergoing reforms to modernize curriculum and teaching methodologies to enhance the quality of medical training and encourage innovative and entrepreneurial thinking among future healthcare professionals [13]. Despite these reforms and the growing emphasis on innovation and entrepreneurship in medical education, empirical research examining how Vietnamese health students acquire innovation competency and entrepreneurial capacity, as well as the factors supporting their engagement in healthcare innovation, remains scarce.

This study addresses the empirical gap by assessing the current level of innovation competency and entrepreneurial capacity among Vietnamese health students and identify key factors influencing their innovation competency and entrepreneurial capacity. The findings inform practical recommendations for integrating innovation and entrepreneurship training into medical education in Vietnam, thereby fostering a more innovative and entrepreneurship-driven healthcare workforce.

## Methodology

### Study design and setting

This cross-sectional study was conducted between May 2025 and July 2025 covering more than ten health related universities across Vietnam. The study targeted Vietnamese health students enrolled in various programs by including a diverse sample from various academic years (1–6), majors (Medicine, Dentistry, Traditional Medicine, Preventive Medicine, Nursing, Public Health, Medical Technology Fields, Allied Health Fields) and regions (north, central, south). The survey collected information on students’ abilities at a single point in time and was designed to evaluate innovation competency and entrepreneurial capacity.

### Study participants

Participants were recruited based on the following inclusion criteria: (1) currently studying in a health related academic program in Vietnam; (2) voluntarily agreeing to participate in the study; and (3) having the ability to complete an online self administered questionnaire. The target population included students actively engaged in both theoretical coursework and practical clinical training to ensure a well rounded perspective on entrepreneurial capacity in medical education.

### Data collection instruments

#### Demographic information

We collected information about gender, academic year, major, university, English proficiency, geographical location and major.

#### Innovation competency

The innovation competency was assessed using a validated instrument developed by Chell & Athayde (2009)[14]. The Youth Innovation Skills Measurement Tool was originally developed for a general youth population, however its core domains, such as creativity, leadership, energy, self-efficacy, and risk-taking, closely align with essential competencies recognized for healthcare professionals. In addition, this study aims to shed light on the perspectives of health students, who represent a young population and the future workforce of technology-driven health systems. The instrument identifies five interrelated components of innovation competency: creativity (6 items), leadership (6 items), energy (7 items), self-efficacy (8 items), and risk propensity (4 items). All items used a 7-point Likert scale ranging from 1 (Strongly Disagree) to 7 (Strongly Agree). Factor analysis and internal consistency of translated versions can be seen in supplement 1.

#### Entrepreneurial capacity

The scale was developed following Turker's (2009) methodological proposal for scale development. In this regard, the first stage of Turker's methodology consists of the production of a set of statements to define indicators of teachable entrepreneurship competencies[15].

Entrepreneurship competencies were assessed across three dimensions: Identification of Opportunities (IDE), Evaluation of Opportunities (EVA), and Exploitation of Opportunities (EXP). Identification of Opportunities was measured using three items adapted from Chandler and Jansen (1992) and Anna et al. (2000)[16, 17]. Evaluation of Opportunities included three items adapted from Tang et al. (2012) [18]. Exploitation of Opportunities was assessed using three items proposed by Bamiatzi et al. (2015) [19].

Management and business competencies consisted of three dimensions: Strategic Competencies (STR), Management Competencies (MAN), and Previous Knowledge and Experience (KNE). Strategic Competencies were measured using five items adapted from Man (2001)[20]. Management Competencies were assessed with six items adapted from Bamiatzi et al. (2015), while Previous Knowledge and Experience included four items derived from Lerner and Almor (2002) [19, 21].

Human resources competencies were assessed through two dimensions: Leadership and Motivation (LMO) and Human Resource Management (HUM). Leadership and Motivation were measured using three items adapted from Bamiatzi et al. (2015), whereas Human Resource Management was assessed with five items originally developed by Mitchelmore and Rowley (2013) [19, 22].

Interpersonal competencies were measured using eight items capturing entrepreneurs’ social competencies, adapted from Man (2001) [20].

The measures of each competency category were obtained through 7-point Likert and semantic differential scales (1 = total disagreement; 7 = total agreement with the proposed statement). Factor analysis and internal consistency of translated versions can be seen in supplement 2.

Entrepreneurship education performance drivers include Entrepreneurship Course, Entrepreneurship Faculty, Entrepreneurship Competition, Entrepreneurship Practice, Entrepreneurship Policy. These factors are adapted from the questionnaires designed based on Entrepreneurship Education Performance of Medical Students (EEPMS) theories [23]. The score for each factor was calculated by summing the scores of its sub-questions, which were measured on a 5-point Likert scale, with five representing strongly agree, four representing relatively agree, three representing average, two representing relatively disagree, and one representing strongly disagree. Factor analysis and internal consistency of translated versions can be seen in supplement 3.

#### Translation of the instruments

The scales were translated from English into Vietnamese by two bilingual medical students, then reviewed by two academic experts in medical education to ensure content validity

### Recruitment and data collection

A convenience sampling strategy was employed. The questionnaire was distributed via Google Forms through medical student groups and professional forums on social media platforms. Participants were informed about the study’s purposes, confidentiality, and voluntary participation before completing the survey. A total of 370 responses were collected from students across various institutions and academic levels.

### Data analysis

All data analyses were carried out using the RStudio software. Descriptive statistics (frequencies, percentages, means, and standard deviations) were used to summarise demographic variables and competency scores. Parametric tests, including independent samples t-tests and one-way ANOVA, were applied to compare group differences due to the normal distribution of data. Linear mixed-effects model analysis (with university as a random effect) was used to identify the related factors with entrepreneurship education performance among health students.

## Results

Table 1 summarizes participants’ distribution across key demographic and educational variables, including region, gender, academic year, professional major, and self-reported English proficiency. Most students were from the Northern (44.9%) and Central (40.3%) regions. The gender distribution was nearly balanced, with 51.9% female and 48.1% male students. Participants represented all six years of training, with the highest proportion in Year 1 (26.2%) and Year 3 (23.5%). The most common majors were general medicine (26.8%), nursing (23.5%), and preventive medicine (16.5%). Regarding English proficiency, the majority reported intermediate levels (B1: 27.0%, B2: 21.9%).

**Table 1:** Demographic characteristics of Vietnamese health science students participating in the study

Categories (*N* = 370)	*N*	%
Gender		
Male	178	48.1
Female	192	51.9
Academic year		
1	97	26.2
2	60	16.2
3	87	23.5
4	61	16.5
5	50	13.5
6	15	4.1
Professional major		
Medicine	99	26.8
Dentistry	33	8.9
Traditional Medicine	45	12.2
Preventive Medicine	61	16.5
Nursing	87	23.5
Public Health	17	4.6
Medical Technology Fields	15	4.1
Allied Health Fields	13	3.5
English proficiency		
A1	60	16.2
A2	90	24.3
B1	100	27.0
B2	81	21.9
C1	27	7.3
C2	12	3.2
Region		
Northern	166	44.9
Central	149	40.3
Southern	55	14.9

Table 2 shows the distribution of innovation competency and entrepreneurial capacity scores among Vietnamese health students. The mean score for innovation competency was 137.06 (SD = 28.91), and entrepreneurial capacity was 173.28 (SD = 35.20). Among the five domains of innovation competency, when standardized to a 0–100 scale, all domains fell within a relatively narrow range of 56 to 58%, indicating a consistent, moderate level of competency. Leadership (58.12) demonstrated the highest standardized score, followed by creativity (57.08), risk propensity (57.04), self-efficacy (56.75), and energy (56.00). The four domains of entrepreneurial capacity, on a standardized 0–100 scale, also exhibited a narrow spread of 55 to 56%, suggesting a uniform, moderate competency level. The highest score was observed in human resources competencies (56.08), while interpersonal (55.60), management and business (55.61), and entrepreneurship (55.07) competencies were slightly lower.

**Table 2:** Distribution of innovation competency and entrepreneurial capacity scores among Vietnamese health students

	Mean (SD)	Min	Max	Standardized score (0-100)
Innovation competency	137.06 (28.91)	31	217	
Creativity	26.55 (6.44)	6	42	57.08
Leadership	31.41 (7.70)	7	49	58.12
Energy	26.16 (6.56)	6	42	56.00
Self-efficacy	35.24 (8.74)	8	56	56.75
Risk propensity	17.69 (4.67)	4	28	57.04
Entrepreneurial capacity	173.28 (35.20)	50	280	
Management and business competencies	65.05 (14.27)	15	105	55.61
Entrepreneurship competencies	38.74 (8.70)	9	63	55.07
Human resources competencies	34.92 (8.55)	8	56	56.08
Interpersonal competencies	34.69 (8.24)	8	56	55.60

Table 3 presents the difference in innovation competency and entrepreneurial capacity scores by gender, academic year, major, English proficiency, and geographical region. Statistically significant differences in Innovation competency were observed by gender (*p* = 0.044), academic year (*p* = 0.001), major (*p* = 0.043), and region (*p* = 0.005). Male students, first-year students and those in general medicine reported the highest scores. Students in the northern region had significantly higher innovation competency scores compared to peers in the central and southern regions. No significant differences were observed based on English proficiency level (*p* = 0.143). Entrepreneurial capacity demonstrated statistically significant variation by gender (*p* < 0.001) and academic year (*p* = 0.008), with male students and those in their first year exhibiting higher scores. No statistically significant differences were identified across major (*p* = 0.372), English proficiency (*p* = 0.214) and geographical region (*p* = 0.567).

**Table 3:** Innovation competency and Entrepreneurial capacity differences among health students

Categories	Innovation competency			Entrepreneurial capacity		
	95%CI	*p*-value	Cohen’s d/${\eta}^{2}$ (95% CI)	95%CI	*p*-value	Cohen’s d/${\eta}^{2}$ (95% CI)
Gender						
Male	135.99–143.68	0.044*	0.12 (0.00–0.24)	175.22–185.07	0.000*	0.23 (0.12–0.34)
Female	130.04–138.92			162.04–172.24		
Academic year						
1	141.89–154.13	0.001*	0.06 (0.02–1.00)	175.38–191.69	0.008*	0.04 (0.01–1.00)
2	126.92–141.78			167.07–183.17		
3	126.93–139.80			160.06–174.88		
4	131.07–141.62			166.97–181.03		
5	121.03–136.53			154.21–175.35		
6	112.13–145.87			149.56–173.64		
Major						
Medicine	136.17–146.68	0.043*	0.04 (0.00–1.00)	168.75–183.37	0.372	0.02 (0.00–1.00)
Dentistry	128.07–141.13			167.27–184.46		
Traditional Medicine	125.10–142.84			165.67–187.49		
Preventive Medicine	128.72–142.95			165.60–181.55		
Nursing	131.88–146.32			162.96–179.80		
Public Health	127.36–163.58			158.86–199.85		
Medical Technology Fields	111.77–133.70			147.94–179.93		
Allied Health Fields	94.80–140.75			127.62–176.99		
English proficiency						
A1	130.03–148.17	0.143	0.01 (0.00–1.00)	154.71–179.19	0.214	0.02 (0.00–1.00)
A2	131.85–145.08			168.15–183.79		
B1	130.60–141.95			168.88–181.30		
B2	134.33–144.68			169.61–182.91		
C1	120.30–137.48			163.35–182.95		
C2	110.87–138.63			137.87–169.14		
Geographical region						
Northern	137.38–146.59	0.005*	0.03 (0.01–1.00)	169.00–181.59	0.567	0.003 (0.00–1.00)
Central	130.13–139.11			167.92–177.33		
Southern	121.74–135.87			161.32–178.20		

* *p* < 0.05

The results of linear mixed-effects model for variables hypothesized to influence entrepreneurship education performance are illustrated in Table 4. Most of the factors were statistically significant with Marginal and Conditional R-squared were 0.324 and 0.598, respectively. Gender exhibited a negative association (β = -0.480), suggesting lower entrepreneurship education performance scores among female students, however this difference was not statistically significant.

**Table 4:** Linear mixed-effects models analysis of factors associated with entrepreneurship education performance among health students

Independent variable (*N* = 370)	Coefficient	Standard Error	*p*-value	*R*_m_²	*R*_c_²
Gender	-0.480	0.276	0.083	0.324	0.598
Entrepreneurship course	0.132	0.067	0.048*		
Entrepreneurship faculty	0.175	0.064	0.006*		
Entrepreneurship competition	0.134	0.073	0.045*		
Entrepreneurship pratice	0.154	0.044	< 0.001*		
Entrepreneurship policy	0.230	0.054	< 0.001*		

* *p* < 0.05

**Fig. 1:**
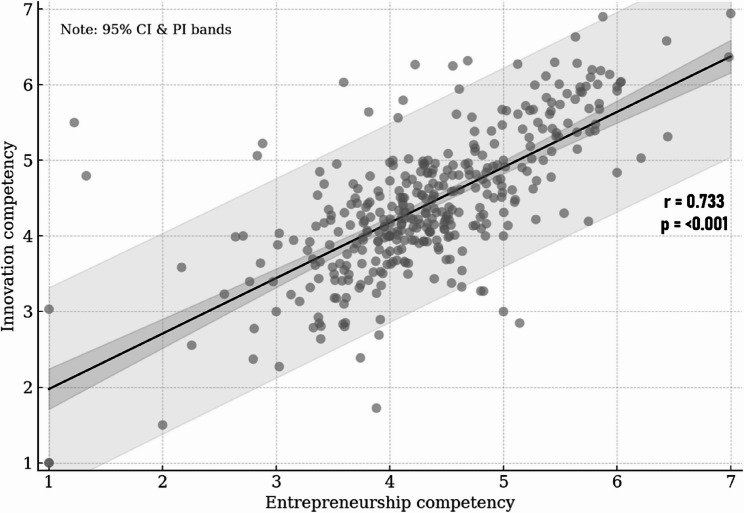
Relationship between innovation competency and entrepreneurial capacity among health students

Figure 1 illustrates a significant positive correlation between innovation competency and entrepreneurial capacity among health students. Each point represents an individual participant, and the fitted linear regression line indicates a strong upward trend (*r* = 0.733, *p* < 0.001). These results support the hypothesis that students with stronger innovation skills tend to report higher levels of entrepreneurial readiness, underscoring the synergistic nature of these two competencies in medical education.

Overall, these results indicate that innovation competency and entrepreneurial capacity are moderately developed, closely interrelated, and shaped by distinct demographic and educational factors.

## Discussion

### Innovation competency and entrepreneurial capacity among health students

Overall, the standardized scores of Vietnamese health students in innovation competency are distributed tightly between 56 and 58 across all five domains. No subdomains (creativity, leadership, energy, self-efficacy, and risk propensity) were particularly strong, indicating only a moderate level of competency. This lack of differentiation suggests that the current educational approach emphasizes general exposure to innovative concepts without focusing on developing confidence in specific skills. Previous research has shown that health professional education in Vietnam is largely clinically oriented, with limited integration of innovation or creativity training into the curriculum [13, 24]. For instance, consistent with the study by Guillen Cuba et al. (2025), which identifies entrepreneurial creativity as the strongest predictor of teaching effectiveness and practical performance in public universities, the present findings suggest that creativity functions as a core driver of innovation [25]. In both contexts, creativity transcends a complementary role and becomes a structural mechanism through which students translate knowledge into applied competencies, particularly in resource-constrained educational environments. International studies also confirm that innovation competency among health students is best enhanced through experiential pedagogies, interdisciplinary teamwork, and problem based learning, which remain underutilized in many Asian contexts [26]. The relatively uniform scores across innovation subdomains therefore reflect structural limitations in curriculum design rather than student motivation, emphasizing the need for systemic transformation. In Vietnam, the higher education innovation ecosystem has expanded in recent years, but systemic barriers, including weak institutional capacity and limited collaboration between university and industry, constrain opportunities for experiential learning such as hackathons, incubators, and interdisciplinary projects [27]. These constraints may also help explain the patterns observed in the present study.

A similar pattern was observed in entrepreneurial capacity, where all four subdomains (management and business, entrepreneurship, human resources, and interpersonal competencies) fell within the narrow range of 55 to 56%. Once again, no domain emerged as a clear strength, pointing to a broad but shallow development of entrepreneurial skills. This finding is consistent with prior work showing that entrepreneurship education in Vietnam and comparable middle-income countries tends to prioritize theoretical management content while underemphasizing opportunity recognition, leadership, and interpersonal competencies, which are crucial for entrepreneurial practice [26, 28]. Furthermore, this limited diversity can be compared with findings from high-income environments, where entrepreneurship programs frequently succeed in developing different qualities, such as opportunity awareness or resilience via simulation-based or competency-based learning and mentorship [29].

The results point to a systemic gap: while Vietnamese students show motivation and potential, the education system lacks mechanisms to build advanced entrepreneurial and innovation skills. Practical initiatives such as experiential programs, incubators, and leadership training could help close this gap and prepare students for a healthcare industry driven by innovation and entrepreneurship in the future.

### Some related factors affect the innovation competency among health students

Our analysis indicated that male students scored higher on innovation competency compared to female peers (*p* = 0.044), highlighting gender as a systemic influence in innovation outcomes. This finding aligns with Alsos et al. (2006), who emphasize that gender within innovation systems yields distinct effects independent of other variables [30]. Moreover, Foss et al. (2013) similarly reported that while women generate ideas as frequently as men, their contributions are less often implemented, reflecting systemic bias rather than differences in ability [31]. These insights suggest that, in the Vietnamese health education context, gender disparities in innovation competency may stem from institutional cultures that privilege male voices. Addressing this requires intentional design of curricula and mentoring structures that amplify female participation, such as gender balanced innovation teams, inclusive leadership training, and targeted support programs.

We found a significant difference across academic years (*p* < 0.001): first-year students displayed higher innovation competency scores than senior cohorts. In a medical student context, early-year learners are often freer from clinical constraints and therefore more willing to engage in exploratory and creative activities. To counteract this attrition, medical schools should consider embedding innovation curricula longitudinally, sustaining exposure through clinical rotations and senior capstone projects to maintain motivation and openness across all years.

No statistically significant differences were detected in innovation competency by English proficiency level (*p* = 0.143). Although prior literature has emphasized the importance of English skills in accessing global knowledge and entrepreneurial networks, our findings suggest that this relationship may be context specific [32, 33]. One possible explanation is that the “information gap” has narrowed substantially in the digital era. Students with lower English proficiency may still demonstrate strong innovation competency by accessing global knowledge through freely available translation tools. Moreover, Vietnam’s entrepreneurial ecosystems are increasingly supported by localized resources and training programs. These findings highlight the need for further research to clarify the contextual pathways through which language skills may influence health students.

Regional differences were pronounced (*p* = 0.005), with students from the Northern region scoring higher in innovation competency than those from the Central and Southern regions. This finding resonates with entrepreneurial ecosystem analyses, such as Vuong (2016), who identified Northern-based entrepreneurs, particularly around Hanoi, as relying more on creative performance in business ventures compared to their Southern counterparts [34]. In Vietnam’s medical training, northern universities often benefit from stronger institutional ties to research centres and incubators based in the capital. Given the northern region’s experience in such development, establishing inter-university collaboration networks and resource-sharing initiatives among regions could help address these disparities. National programs, such as shared mentorship programs, cross-regional startup competitions, and collaborative research projects, may further enhance equitable access to innovation ecosystems and contribute to narrowing regional gaps among health students.

Overall, these findings confirm the need for structural, equity-oriented educational reforms. To foster innovation among all medical students, Vietnamese medical schools would benefit from implementing strategies such as inclusive team-based design projects, sustained innovation curricula across years, enhanced English language and intercultural training, and regional network development. Such interventions can mitigate systemic disparities and support the development of clinician innovators across demographic and geographic lines.

### Some related factors affect the entrepreneurial capacity among health students

Table 3 shows that male students reported significantly higher entrepreneurial capacity than female students in the descriptive analysis (*p* < 0.001). This result is aligned with the analysis of Hechavarría et al. and Zehai Long et al. that there was dissimilarity among men and women in the field [23, 35]. However, this association was no longer statistically significant in the multivariable regression model (Table 4). This discrepancy suggests that the initial gender difference may not be explained by other factors included in the adjusted analysis, such as participation in innovation competitions, entrepreneurship training, or institutional encouragement. In other words, gender itself may not independently reflect entrepreneurial capacity once these contextual and experiential variables are taken into account. By considering the different strengths of each gender, it is possible to better adapt educational methods to different situations. This, in turn, advances the goals of entrepreneurship education.

Both theoretical and practical learning are crucial for growing students’ entrepreneurial knowledge. While entrepreneurship courses provide basic knowledge and skills, practical experiences such as competitions and activities help students to apply what they have learned [36–38]. Through practicals, students may experience the challenges and opportunities of becoming an entrepreneur directly, enhancing their skills and preparedness. A recent cross-sectional study among undergraduates demonstrated that structured learning environments and experiential pedagogical approaches play a critical role in shaping entrepreneurial abilities among health students, supporting the relevance of examining innovation competency within education [39]. Similarly, longitudinal evidence suggests that educational experiences not only influence entrepreneurial intention but also facilitate the transition toward entrepreneurial behaviors, underscoring the importance of competency-based training rather than intention-focused instruction alone [40].

Faculty play a crucial role in this process. Their entrepreneurial knowledge, teaching skills, and personal experience directly influence the effectiveness of entrepreneurial capacity among students [41]. Educators who have both outstanding teaching skills and practical expertise may enhance class relevance by transforming vague concepts into clear, applicable knowledge [42].

### Factors associated with entrepreneurship education performance

Our results show that several factors significantly contribute to entrepreneurship education performance among Vietnamese health students, explaining 59.8% of the variance. Among these, faculty involvement (β = 0.175), practice activities (β = 0.154), and supportive policies (β = 0.230) emerged as the strongest predictors. This aligns with prior research emphasizing that faculty engagement and structured mentorship play a central role in shaping entrepreneurial mindsets and skills [43, 44]. Similarly, entrepreneurship practice provides experiential learning environments that enhance opportunity recognition, risk management, and resilience key capacities often underdeveloped in traditional curricula [45]. Courses (β = 0.132) and competition (β = 0.134) showed relatively weaker associations, suggesting that while formal instruction remains valuable, it is insufficient. This finding supports studies arguing that entrepreneurship education is most effective when complemented by active, real world engagement rather than solely following theoretical modules [46, 47]. Consistent with this perspective, previous studies show that simulation-based and digitally supported teaching methods can effectively strengthen entrepreneurial skills among postgraduate business students, suggesting that similar approaches may also benefit health professions education in increasingly technology-driven settings [48].

Although gender was not a significant predictor in our model, contrasting with evidence from high income countries where gender differences often influence entrepreneurial orientation and performance [49]. Prior research has documented persistent gender gaps in entrepreneurial attitudes, intentions, and resource access, with women frequently reporting lower self-efficacy, higher perceived risk, and differential social expectations compared to men [50]. Other recent work has shown that gender differences in entrepreneurial intent and competencies are not universal but vary by cultural and educational context, with some studies finding no significant differences among students when exposure to entrepreneurship education and others indicating that supportive learning environments can reduce gender gaps in entrepreneurial self-efficacy [51, 52]. These findings point to the role of socialization and structural factors, rather than measurement issues alone. Moreover, this suggests that the absence of a gender effect in our study may reflect a combination of relatively homogenous entrepreneurship exposure among health students, together with the new perceived barriers in the Vietnamese context. Future qualitative research should explore these issues in greater depth.

### Relationship between innovation competency and entrepreneurial capacity

A strong positive correlation (*r* = 0.733, *p* < 0.001) emerged between innovation competency and entrepreneurial capacity, indicating that Vietnamese health students with higher innovation proficiency tend to exhibit stronger entrepreneurial skills. This result aligns with trends observed in Southeast Asia and beyond. For instance, a Malaysian study noted that physician entrepreneurship can directly channel clinicians’ innovative ideas into improved patient care, implying a natural synergy between an innovation mindset and entrepreneurial pursuits [53]. Across Asia, similar relationships are documented: in China, integrating innovation and entrepreneurship training into medical curricula markedly enhanced students’ entrepreneurial capabilities alongside their research competencies, and an Indian survey found a moderate positive association (*r* ≈ 0.51) between students’ innovativeness and entrepreneurial competency [54, 55]. This result aligns with opportunity based entrepreneurship theory, which argues that entrepreneurs succeed by recognizing and exploiting new opportunities within an innovation driven process [26]. In conclusion, the robust innovation competency and entrepreneurial capacity link observed in our Vietnamese cohort not only corroborates regional and global patterns but also underscores that jointly nurturing creativity and entrepreneurial skills in health education may empower future professionals to identify opportunities and drive healthcare innovation.

### Limitations

This study has several limitations that should be considered when interpreting the results. Participants were recruited via social media groups, which may have led to self-selection bias, as students with greater interest in the topic might have been more likely to take part. Additionally, because the survey link was distributed online, the number of students who viewed the invitation but chose not to participate could not be determined, and therefore a response rate could not be calculated. This limits our ability to fully assess the representativeness of the sample. Future studies could address this by using recruitment methods that allow clearer tracking of participation and better control over the sampling process.

## Conclusion

This study offers the first comprehensive assessment of innovation competency and entrepreneurial capacity of Vietnamese health students and the key factors shaping it. While students demonstrated strengths and solid understanding in management and business, self-efficacy and energy, their competencies in opportunity recognition, leadership, interpersonal communication, and especially risk propensity were less developed. From an educational policy and university management standpoint, these findings highlight the need for a structural reorientation of medical education, whereby structured, simulation environments that allow students to experiment and learn from failure, and creativity-driven entrepreneurship training is embedded within curricula. Therefore, fostering an environment where all students, regardless of gender or background, feel empowered to explore and innovate will be key to shaping a more adaptable and entrepreneurial health workforce. Future research should evaluate the impact of targeted interventions and explore how institutional culture, mentorship, and international exposure further influence innovation development.

## Supplementary Information

Supplementary Material 1.

## Data Availability

All data generated or analysed during this study are available on figshare: (10.6084/m9.figshare.30449021.v1).

## Abbreviations

AI: Artificial Intelligence
EVA: Evaluation of Opportunities
EXP: Exploitation of Opportunities
HUM: Human Resource Management
IDE: Identification of Opportunities
KNE: Previous Knowledge and Experience
LMO: Leadership and Motivation
MAN: Management Competencies
SOC: Social Competencies
STR: Strategic Competencies
