# Supplementary material for: Assessing the innovation competency and entrepreneurial capacity of health students in Vietnam: a cross-sectional study
Source: BMC Med Educ. 2026 Mar 17;26:670. doi: 10.1186/s12909-026-08928-y (PMC13107683; doi:10.1186/s12909-026-08928-y)
Supplement: Supplementary file 1 — Supplementary Material 1. [file 12909_2026_8928_MOESM1_ESM.docx]

**SUPPLEMENT**

**Supplement 1. Cronbach's alpha values and factor analysis of translated version of Chell & Athayde’s instrument**

| Factors | Items | Cronbach’s $\alpha$  (95%CI) | Factors loading | |
| --- | --- | --- | --- | --- |
| Creativity | I would like my lessons to involve lots of different creative activities | 0.75  (0.71-0.79) | 0.633 | 0.970 |
|  | I prefer lessons that involve different activities rather than just sitting at my desk |  | 0.654 |  |
|  | I feel proud when I’ve designed something myself and made it |  | 0.752 |  |
|  | I like doing things that are very practical |  | 0.683 |  |
|  | I have chosen subjects at school/college that give me the freedom to express my own ideas |  | 0.628 |  |
|  | The subjects I have chosen at school/college require my imagination |  | 0.628 |  |
| Energy | It’s energising when you are given rewards for good work (e.g. a school day trip) | 0.80  (0.77-0.83) | 0.694 | 0.910 |
|  | I feel really motivated when I produce something that no one else has. |  | 0.748 |  |
|  | I feel really enthusiastic about my chosen subjects. |  | 0.614 |  |
|  | It’s energising and rewarding to help other people. |  | 0.702 |  |
|  | I really push myself to achieve good grades. |  | 0.658 |  |
|  | When I’m doing something I like to feel it has a purpose or goal. |  | 0.718 |  |
|  | I have lots of energy for work and play. |  | 0.653 |  |
| Leadership | I really like being the leader of a group. | 0.79  (0.75-0.82) | 0.720 | 0.914 |
|  | Project work gives me the chance to take a leading role in the group. |  | 0.716 |  |
|  | When working in a group I do my best to persuade the others to take up my ideas. |  | 0.711 |  |
|  | I am often chosen to be the team leader or captain of my team. |  | 0.705 |  |
|  | I like organising other people. |  | 0.664 |  |
|  | My friends follow my suggestions when they can’t make up their minds. |  | 0.671 |  |
| Self-efficacy | I like to pursue my interests outside school/college where I feel more in control. | 0.83  (0.80-0.86) | 0.664 | 0.972 |
|  | I want my future work to be based around a set of challenges that I would find interesting. |  | 0.746 |  |
|  | Once I start something I like to finish it. |  | 0.680 |  |
|  | I would join a club/interest group independently of my friends if it was something I really wanted to do. |  | 0.704 |  |
|  | I’m not easily swayed by other people’s opinions, but do what I think is best. |  | 0.709 |  |
|  | Students should have a say in how a school/college is run. |  | 0.687 |  |
|  | My spending money is important because it gives me a sense of my independence. |  | 0.705 |  |
|  | I’ve been brought up to think for myself |  | 0.655 |  |
| Risk-propensity | When I make choices I want to be as sure as possible what the future consequences will be for me. | 0.74  (0.69-0.78) | 0.744 | 0.949 |
|  | I want my work to provide me with opportunities to show that I can overcome problems. |  | 0.707 |  |
|  | I would not take a risk on an activity that might spoil my chances of getting good grades at school/college. |  | 0.749 |  |
|  | Fearing that I might fail my exams is a powerful motivator at school/college. |  | 0.718 |  |
| Comparative Fit Index (CFI) | | 0.881 | | |
| Tucker-Lewis Index (TLI) | | 0.871 | | |
| Root Mean Square Error of Approximation (RMSEA) | | 0.055 | | |
| Standardized Root Mean Square Residual (SRMR) | | 0.051 | | |

**Supplement 2. Cronbach's alpha values and factor analysis of translated version of entrepreneurship competencies questionnaire**

| **Factors** | **Items** | Cronbach’s $\alpha$  (95%CI) | Factors loading | |
| --- | --- | --- | --- | --- |
| Identification of opportunities (IDE) | I accurately perceive unmet consumer needs | 0.80  (0.76-0.83) | 0.506 | 0.705 |
|  | I spend considerable time and energy looking for products or services that will provide real benefits for my customers |  | 0.413 |  |
|  | One of my greatest strengths is identifying goods and services people want |  | 0.596 |  |
| Evaluation of opportunities (EVA) | I can distinguish between profitable opportunities and not-so-profitable opportunities |  | 0.509 | 0.781 |
|  | I have a knack for telling high-value opportunities apart from low-value opportunities |  | 0.628 |  |
|  | When facing multiple opportunities, I am able to select the good ones |  | 0.552 |  |
| Exploitation of opportunities (EXP) | I am capable of generating creative business ideas |  | 0.669 | 0.772 |
|  | I envision taking advantage of opportunities |  | 0.587 |  |
|  | I am capable of formulating and implementing strategies |  | 0.678 |  |
| Strategic competencies (STR) | I am able to develop and establish longer term directions for the firm, e.g. on the business scale, objetctives, goals or projects | 0.87  (0.85 - 0.89) | 0.601 | 0.765 |
|  | I am able to determine long-term issues, problems, or opportunities |  | 0.642 |  |
|  | I am capable of monitoring progress toward strategic goals |  | 0.582 |  |
|  | I am capable of evaluating results against strategic goals |  | 0.701 |  |
|  | I am able to determine strategic actions by weighing costs and benefits |  | 0.562 |  |
| Management competencies (MAN) | Manage marketing and sales |  | 0.652 | 0.759 |
|  | Manage the financials |  | 0.539 |  |
|  | Develop operational systems |  | 0.601 |  |
|  | Ability to use technology |  | 0.569 |  |
|  | Manage the business |  | 0.622 |  |
|  | Acquire of appropriate resources |  | 0.526 |  |
| Previous knowledge and experience (KNE) | I have some sort of previous entrepreneurial experiences |  | 0.638 | 0.714 |
|  | I am familiar with a certain industry |  | 0.683 |  |
|  | I am familiar with the market |  | 0.665 |  |
|  | I have previous experience managing a business. |  | 0.724 |  |
| Leadership and motivation (LMO) | Leadership skills | 0.84  (0.81- 0.86) | 0.662 | 0.771 |
|  | Motivate others |  | 0.654 |  |
|  | Delegate effectively |  | 0.632 |  |
| Human resources management (HUM) | Employee development |  | 0.606 | 0.861 |
|  | Managing employee performance |  | 0.692 |  |
|  | Human relation management skills |  | 0.662 |  |
|  | Employee relations |  | 0.652 |  |
|  | Hiring skills |  | 0.600 |  |
| Social competencies (SOC) | I'm really good at negotiating with others | 0.82  (0.79 - 0.85) | 0.618 | 0.809 |
|  | I'm really good interacting with others |  | 0.536 |  |
|  | I'm really good at resolving disputes among others |  | 0.597 |  |
|  | I'm really good at maintain a personal network of contacts |  | 0.615 |  |
|  | I'm really good at understand what others mean by their words and actions |  | 0.583 |  |
|  | I'm really good at verbally communicate with others effectively |  | 0.614 |  |
|  | I'm really good at communicating in a written form with others effectively |  | 0.602 |  |
|  | I'm really good at developing long-term trusting relationships with others |  | 0.636 |  |
| Comparative Fit Index (CFI) | | 0.900 | | |
| Tucker-Lewis Index (TLI) | | 0.890 | | |
| Root Mean Square Error of Approximation (RMSEA) | | 0.044 | | |
| Standardized Root Mean Square Residual (SRMR) | | 0.049 | | |

**Supplement 3. Cronbach's alpha values and factor analysis**

**of translated version of**

**Entrepreneurship education performance related questionnaire**

| **Factors** | **Items** | Cronbach’s $\alpha$  (95%CI) | Factors loading |
| --- | --- | --- | --- |
| Entrepreneurship course | Diverse types of entrepreneurship education course | 0.51  (0.38 - 0.56) | 0.426 |
|  | The content of the entrepreneurship course is closely integrated with your own professional knowledge |  | 0.532 |
|  | The content of the entrepreneurship course is closely aligned with the cutting-edge trends of the times |  | 0.473 |
| Entrepreneurship faculty | Teachers teach a variety of styles | 0.61  (0.54 - 0.68) | 0.581 |
|  | Teachers with entrepreneurial experience |  | 0.581 |
|  | Teachers with extensive experience in teaching entrepreneurship education |  | 0.607 |
| Entrepreneurship competition | Variety of entrepreneurship competition | 0.62  (0.56 - 0.69) | 0.569 |
|  | Entrepreneurship competition projects entered are more likely to be landed |  | 0.624 |
|  | High degree of integration of entrepreneurship competition projects with the profession |  | 0.611 |
| Entrepreneurship practice | Entrepreneurship practice with on- and off-campus mentors | 0.8  (0.76 - 0.83) | 0.627 |
|  | Entrepreneurship practice is supported by a dedicated start-up fund |  | 0.592 |
|  | The school offers an integrated entrepreneurship practice service |  | 0.655 |
|  | There is an independent college students pioneer park for entrepreneurship practice |  | 0.614 |
|  | Dedicated off-campus practice base for entrepreneurship practice |  | 0.674 |
|  | High degree of integration of practical entrepreneurship projects with professional studies |  | 0.619 |
| Entrepreneurship policy | State tax relief for university students starting their own businesses | 0.76  (0.72 - 0.80) | 0.662 |
|  | Local governments simplify the application process for university student business registration |  | 0.697 |
|  | The university provides a start-up fund (interest-free loan) for starting a business |  | 0.638 |
|  | Free training from the community to guide your business |  | 0.669 |
| Entrepreneurship education performance | Enriching entrepreneurial knowledge | 0.78  (0.74 - 0.81) | 0.660 |
|  | Cultivating innovation spirit |  | 0.699 |
|  | Improving entrepreneurial skills |  | 0.687 |
|  | Stimulating entrepreneurial intentions |  | 0.687 |
| Comparative Fit Index (CFI) | | 0.891 | |
| Tucker-Lewis Index (TLI) | | 0.872 | |
| Root Mean Square Error of Approximation (RMSEA) | | 0.062 | |
| Standardized Root Mean Square Residual (SRMR) | | 0.054 | |
